# Tension pneumothorax complicating COVID‐19 pneumonia

**DOI:** 10.1002/ccr3.4342

**Published:** 2021-06-24

**Authors:** Amr Mohamed

**Affiliations:** ^1^ Department of Internal Medicine Rochester General Hospital Rochester NY USA

**Keywords:** COVID‐19, pneumothorax

## Abstract

To emphasize the importance of lung imaging in a patient with acute decompensation in the setting of COVID‐19 and to know that pneumothorax is currently a well‐recognized complication related to COVID‐19 pneumonia.

## Graphical abstract

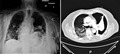

An 83‐year‐old male with recent admission with COVID‐19 pneumonia, during which he completed treatment with dexamethasone and remdesivir and was discharged on 3 L of oxygen. He is now returning to the emergency department with worsening shortness of breath and blood pressure of 60/40. His oxygen saturation on presentation was 60%. His examination was notable for jugular venous distention and no air entry over the left lung.

A chest X‐ray was ordered; however, given that the patient was hemodynamically unstable, the ED physician suspected high‐risk pulmonary embolism(PE), so the patient was rushed to CT before the X‐ray. These imaging modalities showed large left pneumothorax and ground‐glass opacities in the right lung that were present on prior admission consistent with underlying COVID pneumonia as shown in Figure 1.

**FIGURE 1:**
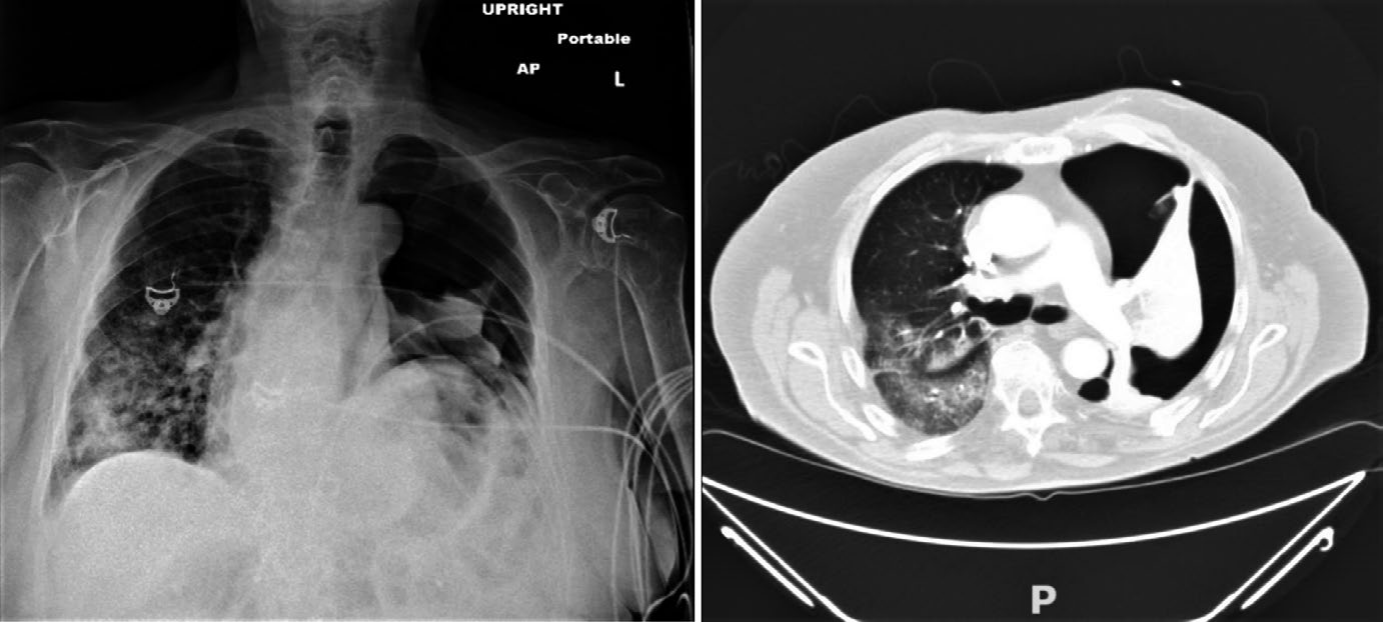
Panel A shows a large left pneumothorax; the left hemidiaphragm is elevated, which is a chronic finding. Panel B is a CT scan of the lung with IV contrast showing large left pneumothorax with lung collapse and showing no evidence of pulmonary embolism; there are ground‐glass opacities in the right lung consistent with known COVID‐19

A chest tube was inserted under complete contact and droplet precautions with a significant improvement in his blood pressure and pneumothorax resolution on follow‐up X‐ray. However, he remained hypoxic, requiring a high‐flow nasal cannula and later BIPAP. Later, the lung infiltrates worsened, and the picture was consistent with re‐worsening of COVID‐19 pneumonia. One of the differentials for his worsening was re‐expansion pulmonary edema**;** however, there were worsening bilateral lung infiltrates rather than isolated worsening of left lung infiltrates. The patient continued to worsen and eventually was converted to comfort care and later died.

The main message is to emphasize that in case of acute decompensation from COVID‐19, we think about worsening ARDS, superimposed bacterial pneumonia, or PE. Diagnosing PE needs a CT scan; not all of us think about pneumothorax, which is frequently reported now ^1^ and only requires a simple chest X‐ray.

## CONFLICT OF INTEREST

None declared.

## AUTHOR CONTRIBUTION

The author contributed to the design and implementation of the research, to the analysis of the results, and the writing of the manuscript.

## ETHICAL STATEMENT

Patient verbal consent had been obtained to use the video material.

## Data Availability

The data that support the findings of this study are openly available in European Respiratory Journal at https://doi.org/10.1183/13993003.02697‐2020. Reference number.^1^
